# Concordance between plasma biomarkers of phosphorylated tau and tau-PET: A narrative review

**DOI:** 10.1177/13872877261468708

**Published:** 2026-08-08

**Authors:** Anna S. Van Houwelingen, Meike W. Vernooij, Marie R. Vermeiren, Rik Ossenkoppele, Elsmarieke Van De Giessen, Harro Seelaar, Julia Neitzel

**Affiliations:** 1 Department of Radiology and Nuclear Medicine, 6993 Erasmus MC, University Medical Centre, Rotterdam, 3000 CA, the Netherlands; 2 Department of Epidemiology, 6993 Erasmus MC, University Medical Centre, Rotterdam, 3000 CA, the Netherlands; 3 685848 Alzheimer Center Amsterdam, Amsterdam UMC, Vrije Universiteit, Amsterdam, the Netherlands; 4 570984 Department of Radiology & Nuclear Medicine, Amsterdam UMC, Vrije Universiteit, Amsterdam, the Netherlands; 5 601873 Amsterdam Neuroscience, Brain Imaging, Amsterdam UMC, Vrije Universiteit, Amsterdam, the Netherlands; 6 647120 Clinical Memory Research Unit, Lund University, Lund, Sweden; 7 Department of Neurology, 6993 Erasmus MC, University Medical Centre, Rotterdam, CA, the Netherlands; 8 569651 Alzheimer Centre, Erasmus MC, University Medical Centre, Rotterdam, CA, the Netherlands

**Keywords:** Alzheimer's disease, blood plasma, positron emission tomography, tau protein

## Abstract

Plasma phosphorylated tau (p-tau) and tau positron emission tomography (PET) are key biomarkers for detecting tau pathology in Alzheimer's disease (AD), offering both diagnostic and prognostic value. However, the concordance between plasma p-tau (p-tau217/p-tau181/p-tau231) positivity and tau-PET positivity remains incompletely understood. In this narrative review, we show that concordance varies by clinical disease stage, the plasma p-tau biomarker assessed, the definition of tau-PET positivity, and the presence of comorbidities and other risk factors. We also present evidence for a temporal sequence of plasma p-tau changes, with plasma p-tau231 positivity preceding p-tau181 positivity, followed by plasma p-tau217 reflecting more advanced stages of tau pathology. We highlight the relevance of these temporal dynamics for the potential development of a stage-specific trajectory framework for plasma p-tau and discuss how such a framework may support improved integration of plasma p-tau and tau-PET, thereby enhancing screening efficiency. Finally, we outline methodological challenges, highlight current efforts such as the development of tau-PET harmonization strategies, and identify priorities for future research. Taken together, this review provides a comprehensive overview of the factors influencing concordance between plasma p-tau positivity and tau-PET positivity. Careful consideration of these factors will be essential for the optimal integration of these biomarkers across the AD spectrum, with the potential to improve both diagnostic and therapeutic strategies.

## Introduction

Recent developments in Alzheimer's disease (AD) research have been marked by advances in biomarkers that reflect key neuropathological processes, such as amyloid-β (Aβ) plaque deposition and tau neurofibrillary tangles.^1^ Among these, biomarkers for detecting in vivo tau pathology show notable diagnostic and prognostic potential, given the strong association between tau accumulation and the severity of cognitive impairment in AD.^2,3^ Tau pathology in AD is characterized by abnormal phosphorylation of tau protein, aggregation into neurofibrillary tangles, and progressive propagation along connected brain regions.^4^ Importantly, available biomarkers capture different aspects of tau pathophysiology: plasma biomarkers of phosphorylated tau (p-tau) are thought to reflect the secretion of soluble p-tau as an early response to Aβ pathology, whereas tau positron emission tomography (tau-PET) detects later-stage neurofibrillary tangles in the brain.^5^

Tau-PET has been shown to accurately distinguish AD from other neurodegenerative diseases and to predict future cognitive decline in both preclinical and symptomatic stages.^6–10^ Moreover, tau-PET provides regional information on tau distribution, and these spatial patterns have been linked to hypometabolism, brain atrophy, and the clinical presentation of different AD subtypes.^11–13^ Despite these clinical advances, tau-PET remains costly, has limited accessibility, and imposes radiation exposure.^14,15^

In recent years, plasma biomarkers of p-tau have emerged as a promising low-cost, accessible, and noninvasive alternative to tau-PET.^16,17^ In contrast to total tau (t-tau), which serves as a nonspecific marker of neuronal injury and is elevated in a variety of conditions such as stroke or head trauma, plasma p-tau biomarkers demonstrate greater specificity for AD-related tau pathology.^18,19^ These include tau phosphorylated at threonine 217 (p-tau217), threonine 181 (p-tau181), and threonine 231 (p-tau231).^18,20,21^ While other plasma biomarkers of tau, including p-tau205, brain-derived tau, and MTBR-tau243, have demonstrated strong correlations with tau aggregates,^22–25^ these markers are less widely validated and adopted, and are therefore beyond the scope of this review.

Notably, plasma p-tau biomarkers have demonstrated comparable performance to their more invasive and costly cerebrospinal fluid (CSF) counterparts in distinguishing AD from non-AD individuals.^18^ Among these, plasma p-tau217 has shown particularly high accuracy in detecting in vivo tau pathology,^26,27^ and in discriminating AD from other neurodegenerative diseases.^18,28^ This is also reflected by the larger fold-changes of p-tau217 relative to p-tau181 and p-tau231, observed when comparing individuals with AD dementia to cognitively unimpaired (CU) elderly.^29–31^ Moreover, while p-tau231 appears to plateau with increasing Aβ deposition in preclinical AD, p-tau217 continues to rise with disease progression.^29^ This longitudinal increase in p-tau217 has been linked to brain atrophy and greater cognitive decline over time.^29,32^ Nevertheless, p-tau181 and p-tau231 have also been shown to reflect tau neurofibrillary tangles and to distinguish individuals with AD from both Aβ-negative CU individuals and other neurodegenerative diseases.^20,31,33^ In addition, higher levels of p-tau181 have been associated with steeper cognitive decline and increased risk of progression from mild cognitive impairment (MCI) to AD.^34,35^

However, plasma p-tau biomarkers also have several limitations compared to tau-PET. While tau-PET captures the aggregation of tau neurofibrillary tangles in the brain, plasma p-tau reflects soluble hyperphosphorylated tau and does not provide regional information on tau deposition.^36,37^ This regional information provided by tau-PET is particularly relevant, as patterns of tau deposition have been shown to vary across clinical phenotypes of AD, including typical amnestic AD, posterior cortical atrophy, and logopenic progressive aphasia.^12,38^ In addition, since plasma p-tau must cross the blood-brain barrier before it appears in plasma, its concentrations are lower and less representative of the entire dynamic range of cortical tau.^37^ Moreover, plasma p-tau has been shown to reflect both tau and Aβ pathology, making it a less specific marker for tau.^39^ Specifically, increases in plasma p-tau have been closely linked to Aβ accumulation, preceding tau-PET positivity.^40^ Overall, plasma p-tau may be considered a proxy for cortical tau pathology, whereas tau-PET provides a more direct and spatially resolved measure of cortical tau deposition and is sensitive to longitudinal changes in tau burden.^36,37,39^

It has been suggested that these differences in the underlying pathophysiology captured by plasma p-tau and tau-PET may help explain discrepancies in biomarker status, depending on the specific biomarker used and the clinical stage assessed.^41,42^ Currently, the concordance between plasma p-tau biomarkers and tau-PET remains incompletely understood. Understanding this concordance is crucial for interpreting and applying these measures, either independently or in combination.^17,27,37^ Therefore, this review aims to assess the concordance between plasma p-tau (p-tau217/p-tau181/p-tau231) positivity and tau-PET positivity. This knowledge will help determine in which contexts plasma p-tau might serve as a substitute for tau-PET, when tau-PET offers unique value, and how both biomarkers can best complement each other in clinical and research settings.

The assessment of the concordance between plasma p-tau positivity and tau-PET positivity is timely, given the potential of these biomarkers for early and accurate diagnosis of AD and improved clinical trial recruitment.^26,27,37^ This timeliness is further highlighted by recent approval of the tau-PET tracer [^18^F]-flortaucipir by the U.S. Food and Drug Administration (FDA) and the European Medicine Agency (EMA) and the FDA clearance of the Lumipulse test (p-tau217/Aβ-42 plasma ratio) to aid in AD diagnosis.^43–45^ Furthermore, the advent of disease-modifying therapies further emphasizes the importance of a comprehensive understanding of these biomarkers to guide treatment decisions, assess treatment effects, and optimize patient management.^16,26,27^

## Methods

We performed a targeted literature search of PubMed using combinations of the keywords “blood biomarker” OR “plasma biomarker” AND “tau PET”, considering publications from January 2003 to February 2026. To broaden the scope, we also reviewed articles listed under the “Similar articles” and “Cited by” sections in PubMed. Plasma biomarkers considered in this review included plasma p-tau217, p-tau181, and p-tau231. Only studies reporting head-to-head comparisons between tau-PET and one or more of these plasma p-tau biomarkers were included to assess concordance. This resulted in the inclusion of 12 studies for concordance assessment. Moreover, for each included study, we extracted: (1) the type of plasma and imaging biomarker(s) assessed; (2) how tau-PET positivity was defined (including the regions of interest and thresholding methods used); (3) the cohort type and name; (4) sample size; and (5) main outcomes and findings. To facilitate structured comparison, studies were grouped according to the specific biomarkers evaluated.

## Concordance between plasma p-tau positivity and tau-PET positivity

Several studies have aimed to assess the concordance between plasma p-tau (p-tau217/p-tau181/p-tau231) positivity and tau-PET positivity. Below, we summarize key findings from studies that compared tau-PET with one or more of these plasma p-tau biomarkers. Key findings are also presented in Table 1.

**Table 1.** Summary of concordance between plasma p-tau positivity and tau-PET positivity across studies.

Cohort	Tau-PET and plasma p-tau markers	Definition of T + (PET)	Main finding
TRIAD, WRAP (Ashton et al.^26^) N = 786 (CU and CI)	[^18^F]MK6240 p-tau181 p-tau217 p-tau231	Temporal meta-ROI (entorhinal cortex, hippocampus, fusiform, parahippocampal, inferior temporal, and middle temporal cortices) 2 SD above mean SUVR of CUY (age <25, defined prior;^46^) SUVR > 1.24 (TRIAD) and SUVR > 1.3 (WRAP)	Plasma p-tau217 (AUC = 0.95 (TRIAD); 0.93 (WRAP)) outperformed p-tau181 (AUC = 0.86; 0.86) and p-tau231 (AUC = 0.80 (WRAP)) for predicting tau-PET status.
UCSF Memory and Aging Center, ARTFL (Thijssen et al.^31^) N = 593 (MCI, AD, FTLD)	[^18^F]flortaucipir p-tau181 p-tau217	Temporal meta-ROI (bilateral entorhinal, amygdala, fusiform, inferior and middle temporal cortices) Youden Index–derived cutoff for distinguishing AD from non-AD neurodegenerative disorders (defined prior;^7^) SUVR > 1.27	Stronger association between tau-PET signal in the temporal cortex and plasma p-tau217 compared to p-tau181. Higher AUC for p-tau217 (AUC = 0.96) than for p-tau181 (AUC = 0.94, Delong test: p = 0.034) in distinguishing tau-PET positive from tau-PET negative individuals.
TRIAD (Ferreira et al.^47^) N = 138 (CU), 87 (CI)	[^18^F]MK6240 p-tau181 p-tau217+p-tau231	Braak-like regions 2.5 SD above mean SUVR of CUY in Braak I region or above (defined prior;^10^) SUVR cut-offs: Braak I = 1.16, Braak II = 1.23, Braak III = 1.55, Braak IV = 1.39, Braak V = 1.56, Braak VI = 1.7	CU: No plasma biomarker provided additional information to demographics (age and sex) to identify tau-PET positivity. CI: p-tau217+ (AUC = 0.93, 95% CI: 0.87–0.99) and p-tau231 (AUC = 0.80, 95% CI: 0.69–0.92) significantly contributed to demographics (AUC = 0.60) to identify tau-PET positivity, while p-tau181 did not.
UCSF Alzheimer Disease Research Center (Mundada et al.^17^) N = 87 Aβ+ (53 MCI, 44 AD)	[^18^F]flortaucipir p-tau217	Continuous SUVR	Plasma p-tau217 concentrations positively associated with tau-PET SUVR in the temporoparietal and dorsolateral prefrontal cortices. Moderate Aβ-PET burden: plasma p-tau217 concentrations most strongly associated with tau-PET SUVR in the temporal cortex. High Aβ-PET burden: plasma p-tau217 concentrations most strongly associated with tau-PET SUVR in primary cortices.
TRIAD, Primary care setting cohort, Neuropathology cohort (Ashton et al.^33^) N = 313 (32 CUY, 159 CU elderly, 54 MCI, 42 AD, 26 non-AD)	[^18^F]MK6240 p-tau181 p-tau231	Braak-like regions Regional SUVR in the transentorhinal (stage I–II), limbic (III–IV), and isocortical (V–VI) regions 2.5 SD above mean SUVR of CU Aβ-negative elderly	Plasma p-tau231 was highly correlated with tau-PET. The inflection point of plasma p-tau231 preceded thresholds of Aβ-PET positivity and plasma p-tau181 increase. Plasma p-tau231 differentiated individuals across Braak stages (including Braak 0 through Braak I–II) which was not observed for plasma p-tau181.
MCSA or Alzheimer's Disease Research Center (Jack et al.^1^) N = 1136 (864 CU, 272 CI)	[^18^F]flortaucipir p-tau181	1) Topographic staging Braak-like regions 2 SD above mean SUVR of normal group (Gaussian mixture model) SUVR cut-offs: Braak I–II = 1.28, Braak III–IV = 1.33, Braak V–VI = 1.30 2) Magnitude staging Temporal meta-ROI (entorhinal cortex, amygdala, fusiform, parahippocampal, inferior temporal gyrus and middle temporal gyrus) Two cut-off points: SUVR = 1.29 (defined by a neuropathological standard,^48^) SUVR = 1.43 (lower quartile Braak III–IV) Three groups: T^LOW^, T^INT^, T^HIGH^	CU: Adding plasma p-tau181 concentrations to the base model (age, sex, and *APOE* ε4 genotype) improved discrimination between Braak stages 0 and I–II, and between T^LOW^ and T^INT^ tau-PET magnitude stages. CI: Addition of plasma p-tau181 enhanced discrimination between Braak stages 0 and I-II, and between Braak stages III–IV and V–VI. CU and CI combined: Addition of plasma p-tau181 improved discrimination between Braak stages 0 and I–II, between T^LOW^ and T^INT^, and between T^INT^ and T^HIGH^ tau-PET magnitude stages. Models with p-tau181 provided similar discrimination of tau-PET stages to models with four plasma analytes (p-tau181, Aβ-ratio, GFAP and NfL)
TRIAD (Tissot et al.^37^) N = 284 (30 CUY, 162 CU, 60 MCI, 32 AD)	[^18^F]MK6240 p-tau181 p-tau231	Temporal meta-ROI (entorhinal, amygdala, fusiform, inferior and middle temporal cortices) 2 SD above mean SUVR of CUY (age <25, defined prior;^46^) SUVR > 1.24	Concordance: 82% of plasma p-tau181/PET, 76% of plasma p-tau231/PET and 75% of p-tau231/p-tau181. Plasma p-tau231 positivity may precede p-tau181 positivity.
BioFINDER-2 (Warmenhoven et al.^27^) N = 998 (514 CU, 284 CI)	[^18^F]RO948 p-tau217 Mass spectrometry: %p- tau217_WashU_ p-tau217_WashU_ Immunoassays: p-tau217_Lilly_ p-tau217_Janssen_ p-tau217_ALZpath_	Temporal meta-ROI 2.5 SD above mean SUVR of Aβ-negative young controls in Braak I–VI (defined prior;^49^) ≥1.362 SUVR	All plasma p-tau217 tests exhibited a high ability to detect abnormal tau-PET (AUC range: 0.94–0.97). Plasma %p-tau217_WashU_ had the highest performance and exhibited stronger associations with baseline and longitudinal tau-PET compared with immunoassays. Immunoassays: plasma p-tau217_Lilly_ was more associated with tau-PET load than plasma p-tau217_Janssen_ and plasma p-tau217_ALZpath_.

For each study, the table includes the tau-PET and plasma p-tau markers assessed, the definition of tau-PET positivity (T+), and the main findings regarding the performance of plasma p-tau markers in predicting tau-PET status. Definition of T + (PET): specifies the region of interest (ROI), the method used to define thresholds, and the thresholds applied to determine tau-PET positivity. Note: Several studies include the TRIAD cohort, although specific subsamples differ according to inclusion periods, and definitions of T + (PET) vary across studies.

AD: Alzheimer's disease; ARTFL: Advancing Research and Treatment for Frontotemporal Lobar Degeneration Consortium; AUC: area under the curve; CI: cognitively impaired; CU: cognitively unimpaired; CUY: cognitively unimpaired young; FTLD: frontotemporal lobar degeneration; MCI: mild cognitive impairment; MCSA: Mayo Clinic Study of Aging; ROI: region of interest; SD: standard deviation; SPIN: Sant Pau Initiative on Neurodegeneration cohort; SUVR: standardized uptake value ratio; TRIAD: Translational Biomarkers in Aging and Dementia cohort; UCSF: University of California San Francisco; WRAP: Wisconsin Registry for Alzheimer's Prevention cohort.

A cohort study using data from three observational cohorts found that plasma p-tau217 outperformed both p-tau181 and p-tau231 in predicting tau-PET status.^26^ In addition, plasma p-tau217 effectively distinguished individuals with abnormal tau-PET among Aβ-positive participants, and its concentration increased with advancing Braak stages as assessed by tau-PET.^26^ These findings are consistent with previous research showing a higher area under the curve (AUC) for p-tau217 in distinguishing tau-PET positive from tau-PET negative individuals,^31^ as well as a stronger association between tau-PET signal in the temporal cortex and plasma p-tau217 compared to p-tau18.

Interestingly, Ferreira et al. (2023) not only assessed the association between plasma p-tau concentrations and tau-PET positivity but also examined the relationship between plasma p-tau, entorhinal tau-PET standardized uptake value ratio (SUVR) values, and voxel-wise tau-PET patterns. In CU individuals, none of the plasma biomarkers, p-tau217, p-tau181, or p-tau231, significantly improved a demographic model for predicting tau-PET positivity.^47^ In contrast, in cognitively impaired (CI) individuals, the addition of p-tau217 and p-tau231 significantly enhanced the model's predictive performance for tau-PET positivity.^47^ Further analysis revealed that in CU individuals, entorhinal tau-PET SUVR values were significantly associated with plasma p-tau217 and p-tau231 concentrations, with p-tau231 accounting for the most variance.^47^ Among CI individuals, SUVR values were significantly associated with plasma p-tau217, p-tau181, and p-tau231 concentrations, with p-tau217 explaining the greatest variance, followed by p-tau231.^47^ Voxel-wise analysis supported these findings. In CU individuals, plasma p-tau217 and p-tau231 concentrations were positively associated with tau-PET SUVR values in the precuneus and temporal lobe.^47^ In CI individuals, these biomarkers were associated with SUVR values across the entire cerebral cortex, while p-tau181 was associated with SUVR values in the precuneus and temporal cortex.^47^ These findings highlight that plasma p-tau biomarkers reflect tau deposition in both early and late Braak stages, indicating distinct biomarker properties across different disease stages.

A similar regional approach was employed by Mundada et al. (2023), who found that in Aβ-positive individuals with MCI or AD dementia, plasma p-tau217 concentrations were positively associated with tau-PET SUVR values in the temporoparietal and dorsolateral prefrontal cortices. Furthermore, when stratifying by Aβ-PET burden, plasma p-tau217 concentrations were most strongly associated with tau-PET SUVR values in the temporal cortex among individuals with moderate Aβ-PET burden, whereas in those with high Aβ-PET burden, p-tau217 showed the strongest association with tau-PET SUVR values in primary cortices.^17^ With higher Aβ-PET burden, tau pathology in the temporal cortex is likely more saturated, resulting in a reduced association between plasma p-tau217 concentrations and tau-PET SUVR values in this region. The authors highlight that this spatial pattern aligns with the progression of tau pathology across Braak stages, which typically advances from the medial temporal lobes to primary cortices.^17,50^

Despite the demonstrated superior performance of plasma p-tau217 over p-tau181 and p-tau231,^26,31^ several studies have also reported concordance between tau-PET and both p-tau181 and p-tau231.^1,33,37^ Jack et al. (2023), for example, used both a tau-PET topographic stage, reflecting Braak stages 0, I–II, III–IV, and V–VI, and a tau-PET magnitude stage, categorized as none/low, intermediate, or high, to assess whether plasma p-tau181 concentrations improved discrimination between these tau-PET stages when added to a base model. Among CU individuals, adding plasma p-tau181 concentrations improved discrimination between Braak stages 0 and I–II, as well as between the none/low and intermediate tau-PET magnitude stages.^1^ Among CI individuals, p-tau181 enhanced discrimination between Braak stages 0 and I–II, and between Braak stages III–IV and V–VI.^1^ When considering CU and CI individuals combined, p-tau181 improved discrimination between Braak stages 0 and I–II, between none/low and intermediate tau-PET stages, and between intermediate and high tau-PET stages.^1^

In contrast to studies examining continuous plasma p-tau concentrations, Tissot et al. (2022) assessed dichotomized biomarker status concordance between plasma p-tau181, p-tau231, and tau-PET. This approach yielded four possible groups: concordant positive (p-tau^+^/tau-PET^+^), concordant negative (p-tau^–^/tau-PET^–^), and two discordant groups; p-tau^+^/tau-PET^–^ and p-tau^–^/tau-PET^+^.^37^ The majority of individuals exhibited concordant biomarker statuses, with 82% concordance between plasma p-tau181 and tau-PET, and 76% concordance between plasma p-tau231 and tau-PET.^37^ Among discordant p-tau^+^/tau-PET^–^ cases, individuals were primarily CU and Aβ-positive or CI and Aβ-positive, potentially reflecting early stages of AD in which tau pathology has not yet reached the threshold for tau-PET positivity.^37^ Moreover, plasma p-tau181 and p-tau231 statuses were concordant in 75% of individuals, with plasma p-tau231^+^/p-tau181^–^ cases being more common than plasma p-tau231^–^/p-tau181^+^ among the discordant pairs.^37^ Given that the latter group primarily consisted of CI individuals, and that p-tau231^+^/tau-PET^–^ was more prevalent than p-tau181^+^/tau-PET^–^, the authors suggested that plasma p-tau231 positivity may precede p-tau181 positivity.^37^ This pattern aligns with findings from Ashton et al. (2021), who also reported that plasma p-tau231 increases earlier than p-tau181 and may reflect early tau deposition. Together, these findings suggest a temporal sequence in plasma p-tau biomarker changes and a potential temporal dissociation between plasma biomarkers and tau-PET, which will be further explored in the following section.

## Temporal dynamics of plasma p-tau and tau-PET concordance

Evidence for a temporal sequence in plasma p-tau biomarker changes has emerged from several studies. For instance, Ashton et al. (2021) demonstrated that plasma p-tau231 levels increase with Aβ accumulation, even before the threshold for Aβ-PET positivity is reached, and precede elevations in plasma p-tau181. Moreover, plasma p-tau231 showed the strongest association with tau-PET signal in the entorhinal cortex (Braak I–II), consistent with early tau deposition, and effectively distinguished between early and late Braak stages of tau accumulation.^33^ Specifically, plasma p-tau231 was significantly elevated in Braak I–II compared to Braak 0, a distinction not observed for plasma p-tau181.^33^ Additional support for plasma p-tau231 as an early marker of AD pathology comes from neuropathological studies showing that phosphorylation at threonine 231 occurs earlier than at other tau phosphorylation sites, which have been reported to exhibit significant increases only at Braak stages V–VI.^51,52^

In contrast, associations between plasma p-tau181 and tau-PET appear to depend on the presence of Aβ pathology.^2^ Among individuals with subjective cognitive decline (SCD), plasma p-tau181 was significantly associated with tau-PET signal in Aβ-positive individuals, but not in those who were Aβ-negative.^2^ Stratified analysis by clinical stage further revealed a moderate association between plasma p-tau181 and tau-PET in SCD, and a weak to moderate association in individuals with MCI or AD dementia.^2^ These findings suggest that plasma p-tau181 may also serve as an early marker of tau pathology, but primarily in the context of significant Aβ accumulation.

Interestingly, plasma p-tau217 was shown to be associated with both Aβ-PET and tau-PET, depending on the disease stage.^53^ In early stages of AD, when tau-PET was still negative, plasma p-tau217 was associated exclusively with Aβ-PET.^53^ However, in later stages, when both Aβ-PET and tau-PET were positive, plasma p-tau217 was associated with both biomarkers, with the strongest association observed for tau-PET.^53^ Among individuals with high Aβ-PET uptake, higher plasma p-tau217 concentrations were associated with higher tau-PET SUVR values.^53^ Furthermore, plasma p-tau217 mediated the effect of Aβ-PET on tau-PET uptake, with the strongest effect observed outside the medial temporal lobe.^53^ These findings were supported by postmortem neuropathological data, which demonstrated a similar mediation effect of plasma p-tau217 on the relationship between Aβ plaque density and tau tangle burden, particularly in the neocortex.^53^ Collectively, these results suggest that plasma p-tau217 may play a role in the progression of tau pathology beyond the medial temporal lobe.^53^

One possible explanation for these temporal dynamics is that they may reflect site-specific phosphorylation driven by distinct upstream kinase activities.^40,54^ Tau contains numerous phosphorylation sites regulated by kinases such as GSK-3β, CDK5, and MAPKs, which exhibit context-dependent activity and may respond with varying sensitivity to Aβ pathology.^40,55^ While such differential kinase activation could contribute to a temporal ordering of tau phosphorylation, the extent to which this explains plasma p-tau biomarker dynamics remains to be explored.

Whereas plasma p-tau biomarkers primarily reflect early tau-related changes in AD, tau-PET positivity represents tau pathology at more advanced disease stages.^7,42^ This distinction is supported by findings showing that tau-PET had lower discriminative accuracy in individuals with MCI compared to those with AD dementia.^7^ Moreover, tau-PET positivity has been reported to correspond to Braak stage IV or higher, further suggesting that detectable signal changes occur predominantly after substantial tau spread.^48,56^

**Figure 1.**
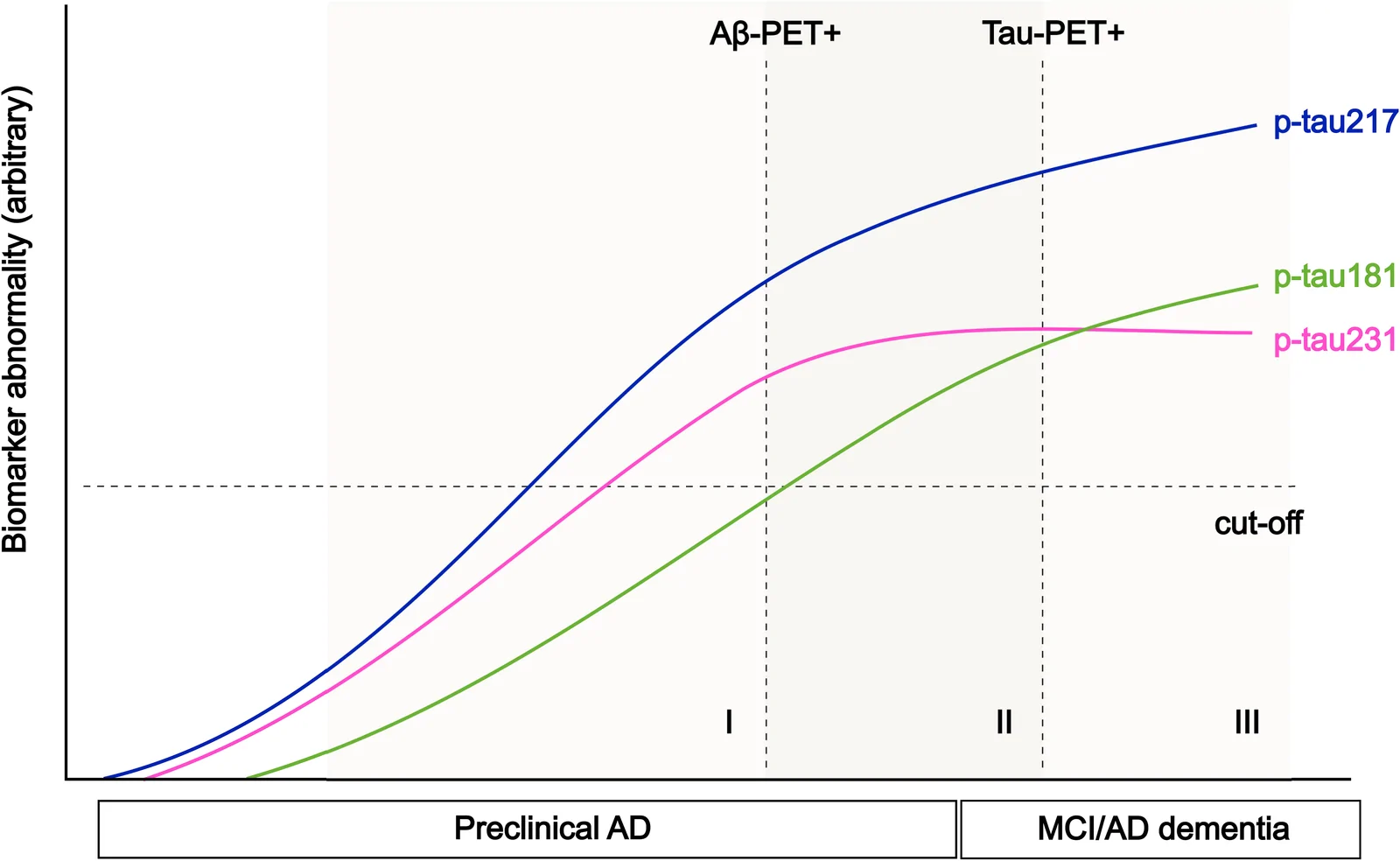
Proposed temporal sequence of plasma p-tau changes across the AD continuum. Plasma p-tau231 changes occur early in disease progression, before Aβ-PET positivity is reached (plane I), and plateau as Aβ load increases (plane II).^33^ Plasma p-tau181 changes emerge with Aβ accumulation (plane II).^2^ Plasma p-tau217 changes are associated with Aβ-PET in early stages (plane I and II) and with tau-PET in later stages (plane III), rising with disease progression.^53^ Note: The sequence is derived from previous literature, and biomarker changes depicted in the figure are arbitrary. AD: Alzheimer's disease; MCI: mild cognitive impairment.

Taken together, differences in concordance between plasma p-tau biomarkers and tau-PET across clinical disease stages may be explained by a temporal sequence in plasma p-tau changes and by differences in sensitivity to early versus late tau deposition.^33,42,53^ Specifically, a temporal dissociation between these biomarkers may exist in preclinical stages, during which plasma p-tau levels begin to rise prior to detectable changes on tau-PET.^37,47,53^ In contrast, greater concordance is typically observed in symptomatic stages, when both plasma p-tau and tau-PET measures are positive.^37,47,53^ Collectively, these findings underscore that biomarker concordance is highly context-dependent and highlight the importance of interpreting biomarker levels within the framework of clinical disease stage (Figure 1).^57^

## Combined use of plasma p-tau biomarkers and tau-PET

**Figure 2.**
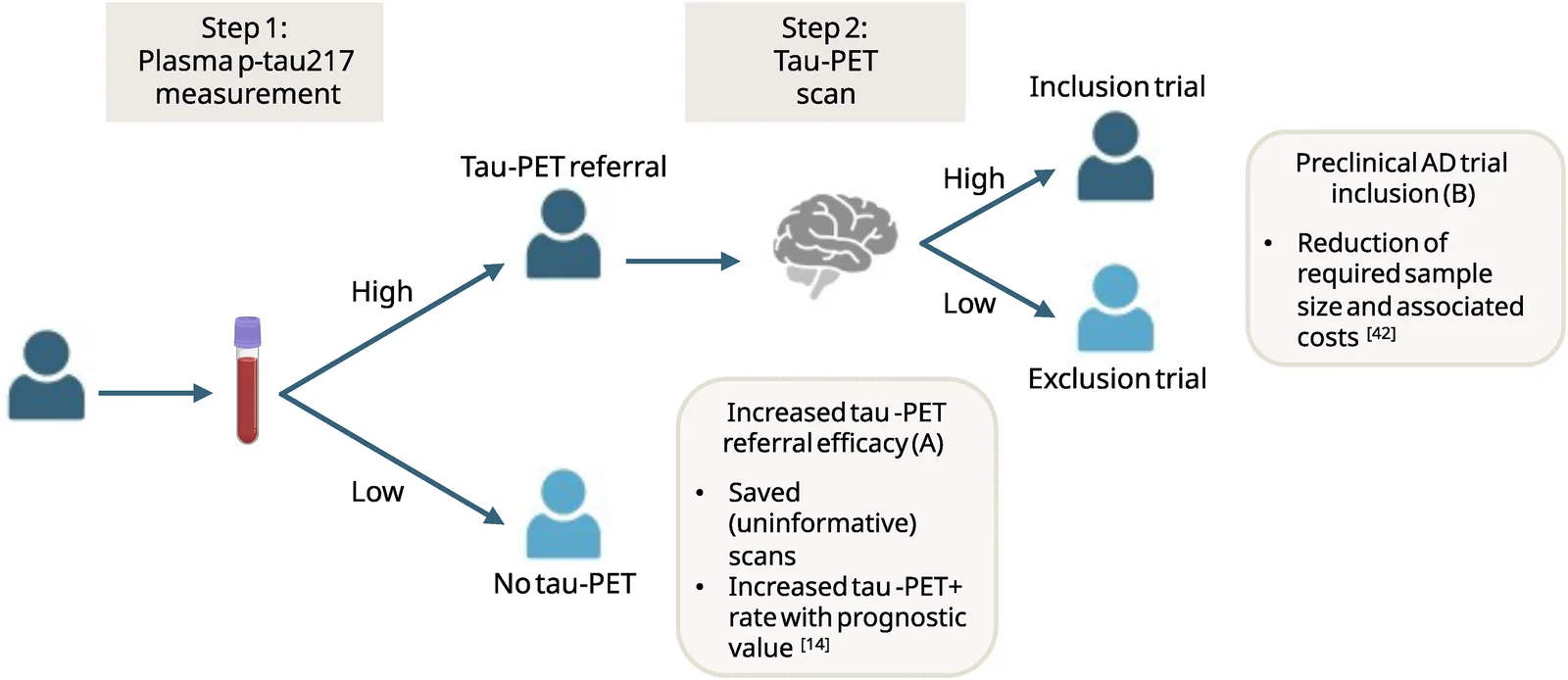
Proposed workflows for the combined use of plasma p-tau217 and tau-PET. Box A illustrates plasma p-tau217 screening for tau-PET referral in memory clinic patients,^14^ whereas Box B illustrates sequential plasma p-tau217 and tau-PET screening for a hypothetical preclinical Alzheimer's disease (AD) trial in cognitively unimpaired individuals with tau tangle pathology.^42^.

Given the differences in the underlying pathophysiology captured by plasma p-tau and tau-PET, as well as their respective strengths and limitations, there has been growing interest in how these biomarkers can most effectively complement one another.^14,42^ In particular, the use of plasma p-tau biomarkers as triage tests has been proposed, whereby a positive plasma p-tau result must be followed by a confirmatory test, such as PET or CSF.^27,58^ Additionally, a dual cut-off strategy has been suggested, in which only individuals with plasma p-tau values within an intermediate range, between the lower and upper thresholds, undergo confirmatory testing.^59^ The majority of studies to date have focused on the combined use of plasma p-tau217 and tau-PET, which we summarize here (Figure 2).

In memory clinic patients, Brum et al. (2024) evaluated whether plasma biomarkers could serve as a screening tool to optimize tau-PET referrals. Cut-offs for plasma p-tau217, p-tau181, and p-tau231 were established to detect tau-PET positivity, and referral efficiency was assessed by the percentage of saved scans and the tau-PET positivity rate among those above the threshold.^14^ Across both metrics, plasma p-tau217 outperformed the other plasma biomarkers: at a 95% sensitivity cut-off, plasma p-tau217 saved nearly 50% of scans and achieved a tau-PET positivity rate of approximately 70%, significantly higher than that observed in the overall study population (37%).^14^ Importantly, tau-PET was associated with future cognitive decline only among individuals above the p-tau217 threshold, underscoring the prognostic value of this workflow.^14^ Moreover, the authors emphasize that assessment of regional tau deposition in referred individuals may help differentiate between AD subtypes.^13,14^ These findings demonstrate the clinical utility of a two-step workflow using plasma p-tau217 as an initial screening tool to optimize tau-PET referrals.

A similar two-step approach was employed by Ossenkoppele et al. (2025), who assessed whether sequential use of plasma p-tau217 followed by tau-PET could reduce the required sample size for a hypothetical preclinical AD trial, using cognitive decline on the modified preclinical Alzheimer cognitive composite (mPACC5) as the outcome. Restricting inclusion based on plasma p-tau217 quartiles substantially reduced the required sample size: selection of quartiles 2–4, 3–4, and 4 yielded reductions of 32%, 64%, and 82%, respectively.^42^ Applying tau-PET as a second step led to additional reductions. For instance, among participants in quartiles 3–4 of plasma p-tau217, further restricting inclusion to those with tau-PET levels in quartile 4 increased the reduction in sample size from 64% to 88%.^42^ Furthermore, four groups were defined based on varying combinations of plasma p-tau217 and tau-PET thresholds to estimate the cost savings that could be achieved through the proposed two-step screening approach, assuming the cost of a tau-PET scan to be equivalent to approximately 15 plasma assessments.^42^ The p-tau217 quartile 4 only group and the group with both plasma p-tau217 and tau-PET restricted to quartile 4 yielded substantial cost reductions of 96.9% and 96.6%, respectively. The moderate and liberal groups yielded cost reductions of 85.6% and 54.5%, respectively.^42^ Overall, this study demonstrates that the sequential use of plasma p-tau217 followed by tau-PET can enhance screening efficiency for preclinical trials by substantially reducing both the required sample size and associated costs.^42^

A promising direction for future research is to evaluate how plasma p-tau biomarkers and tau-PET can be combined to optimize their utility across different clinical and research contexts.^14^ The optimal application of these biomarkers will likely depend on the intended use: for instance, in prescreening for a trial, a higher false negative rate may be acceptable, whereas in diagnostic settings, minimizing false negatives is critical. Moreover, optimal use may vary by disease stage, reflecting the biomarkers’ differing associations with cognitive decline.^2,42^ The relationship between plasma p-tau, tau-PET, and clinical outcomes will be discussed in the following section.

## Plasma p-tau biomarkers and tau-PET in relation to clinical outcomes

Determining the extent to which plasma p-tau biomarkers and tau-PET predict future cognitive decline and disease progression is critical for both clinical trial recruitment and patient management.^2,42^ This section summarizes findings from studies that have conducted head-to-head comparisons of plasma p-tau biomarkers and tau-PET in predicting these clinical outcomes.

In a sample of CU individuals, Ossenkoppele et al. (2025) examined the individual and combined associations of plasma p-tau217 and tau-PET with cognitive decline on the mPACC5, as well as with future progression to MCI. In separate models, both plasma p-tau217 and tau-PET significantly predicted cognitive decline with similar performance.^42^ When combined in a single model, both biomarkers remained significant predictors, and the joint model outperformed the individual biomarker models, highlighting their complementary value.^42^ Similar results were observed in the Aβ-positive CU subgroup, although tau-PET contributed relatively more to explaining cognitive decline than in the full sample.^42^ A comparable pattern was found for progression to MCI: both plasma p-tau217 and tau-PET were significant individual predictors with similar performance, and the combined model again showed improved predictive accuracy.^42^ Notably, this similar performance between plasma p-tau217 and tau-PET in predicting cognitive decline is generally not observed in CI individuals, where tau-PET has been shown to outperform plasma p-tau217,^17^ consistent with the more advanced stage of tau pathology captured by tau-PET.

This discrepancy was further demonstrated in a study by Mundada et al. (2023), which included Aβ-positive individuals with MCI or AD dementia. Cross-sectionally, both higher plasma p-tau217 and tau-PET SUVR values were independently associated with lower MMSE scores.^17^ Moreover, when combined in a single model, both biomarkers remained significant predictors, and the joint model outperformed the individual models.^17^ However, in predicting cognitive decline, while both biomarkers were individually associated with longitudinal MMSE, only tau-PET remained a significant predictor in the combined model.^17^ This discrepancy between CU and CI individuals may reflect differences in the pathophysiological processes captured by plasma p-tau and tau-PET, and their distinct temporal dynamics, as previously discussed.^42^ These findings suggest that plasma p-tau217 may have greater prognostic value in earlier disease stages, whereas tau-PET may be more informative in symptomatic stages.

A similar pattern of stage-specific biomarker performance has been observed in head-to-head comparisons between plasma p-tau181 and tau-PET.^2^ Specifically, plasma p-tau181 was associated with cognitive decline on the MMSE and the Rey Auditory Verbal Learning Test (RAVLT) delayed recall among individuals with SCD, but not in those with MCI or AD.^2^ In contrast, tau-PET was associated with cognitive decline across all examined neuropsychological tests in individuals with SCD, and was additionally associated with cognitive decline on the MMSE and the Category fluency test (CFT) animals in those with MCI or AD.^2^ Furthermore, although both plasma p-tau181 and tau-PET levels showed greater longitudinal increases in individuals with MCI or AD than in those with SCD, only annual changes in tau-PET, but not p-tau181, were associated with cognitive decline.^2^ Based on these findings, the authors conclude that tau-PET is a more reliable marker for monitoring clinical progression than plasma p-tau181.^2^

Besides the favorable prognostic performance of tau-PET in CI individuals in these head-to-head comparisons, other studies have demonstrated that plasma p-tau biomarkers also provide valuable prognostic information.^34,35^ For instance, plasma p-tau217 and p-tau181 have been shown to accurately predict individual risk of progression from MCI to AD,^34^ and higher levels of plasma p-tau181 have been observed in MCI converters compared to non-converters.^35^ While Palmqvist et al. (2021) provides promising evidence for individual-level prediction using plasma p-tau, most current studies report findings at the group level.^2,17,42^ Therefore, further validation and the development of clinical guidelines are required before these biomarkers can be used for individual-level prognosis in routine clinical practice.^34^ These findings underscore that both plasma p-tau biomarkers and tau-PET offer clinically relevant information, with their predictive utility depending on disease stage, the specific plasma p-tau biomarker used, and whether cognitive outcomes are assessed cross-sectionally or longitudinally.

## Methodological challenges

**Figure 3.**
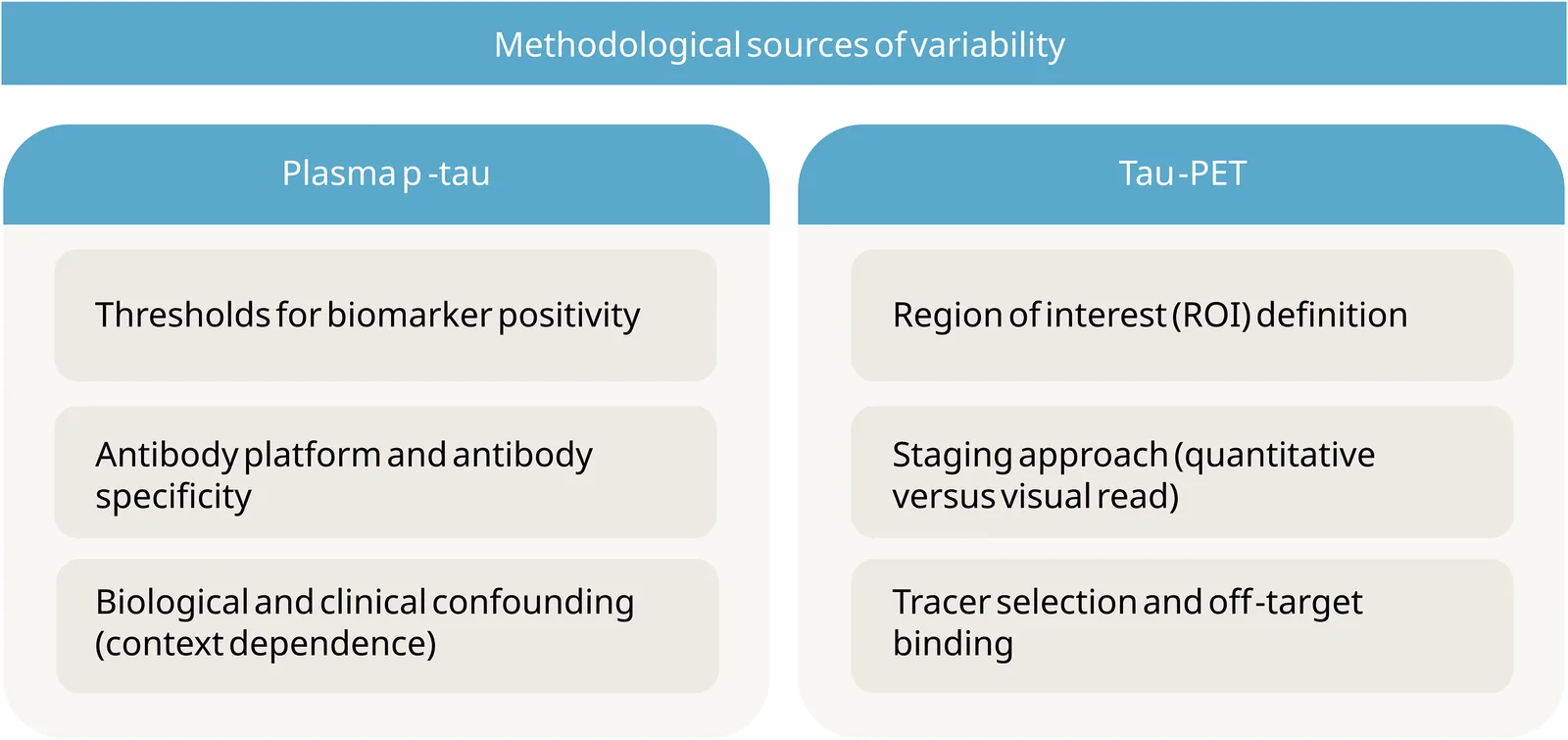
Conceptual overview of key methodological challenges and sources of variability in plasma p-tau and tau-PET relevant to cross-study comparability.

Several methodological challenges are inherent to the use of plasma p-tau biomarkers and tau-PET. These include establishing thresholds for biomarker positivity, selecting an appropriate target region of interest (ROI) for tau-PET, determining a staging scheme (e.g., quantitative versus visual read methods), and accounting for variability across plasma p-tau assays and tau-PET tracers.^10,27,42^ These factors can significantly impact the comparability of findings across studies and warrant careful consideration to ensure the optimal implementation of plasma p-tau biomarkers and tau-PET in both research and clinical settings. In the following sections, we elaborate on several of these methodological challenges (Figure 3).

### Binary classification versus severity-based staging of tau-PET

Tau-PET is often classified in a binary fashion, i.e., tau-PET positivity versus negativity, based on a single cut-off applied to a temporal meta-ROI SUVR.^14,26,27,37^ However, the regions included in this temporal meta-ROI vary across studies,^1,26,37^ partly because current diagnostic guidelines from the National Institute on Aging and Alzheimer's Association (NIA-AA) do not specify exact regions.^24^ In addition, studies differ in the reference groups used to establish biomarker cut-offs. For instance, some studies use CU Aβ-negative older adults,^33^ while others use CU young individuals, given the age-related nature of tau pathology.^26,37^ These variations in ROI selection and reference group definition should be taken into account when interpreting and comparing findings across studies.

Furthermore, given the unique topographic and quantitative information provided by tau-PET and its correlation with clinical phenotypes of AD,^12,38^ several studies emphasize the value of severity-based staging over binary classification.^1,41^ For example, Mattsson-Carlgren et al. (2020) demonstrated that using continuous tau-PET SUVR values improved the prediction of cognitive decline compared to binary classification, underscoring the prognostic utility of preserving continuous biomarker information. Moreover, Jack et al. (2023) highlighted the relevance of severity-based staging for clinical trial recruitment and treatment arm assignment. Specifically, they employed both a topographic Braak-like staging scheme and a magnitude-based approach, using two SUVR cut-offs to categorize individuals into low, intermediate, and high tau-PET groups.^1^ While acknowledging that more fine-grained staging approaches exist, the authors note that these would have resulted in an unmanageable number of pairwise comparisons.^1^ Thus, the key challenge is to adopt a tau-PET staging approach that balances sufficient granularity to retain prognostic information with the statistical power required for practical implementation.

### Visual read methods of tau-PET

In addition to quantitative approaches described above, which provide valuable prognostic and diagnostic information in research settings,^1,41^ tau-PET can also be interpreted using visual read methods, which are essential for feasible clinical application.^60^ These methods rely on qualitative assessment of cortical uptake patterns rather than predefined SUVR thresholds, facilitating standardized interpretation in clinical settings. Whereas Aβ-PET visual read methods have been validated for several FDA-approved tracers,^61^ visual read methods for tau-PET are still undergoing standardization.^60,62^

Currently, [^18^F]-flortaucipir is the only tau-PET tracer approved by the FDA and the EMA,^43,44^ with an accompanying visual read method for clinical use in patients with cognitive impairment evaluated for AD.^60,63^ This visual read method captures late-stage tau pathology corresponding to Braak stages V/VI and has demonstrated excellent inter-reader agreement and strong concordance with SUVR-based classification.^63^ Furthermore, a positive visual read has been associated with future cognitive decline in CU individuals and those with AD.^63^

Nevertheless, findings regarding visual read methods for other tau-PET tracers are also promising.^14,62,64^ For instance, Brum et al. (2024) applied both a previously established SUVR cut-off and a clinically validated visual read method to assess tau-PET positivity using [^18^F]RO948.^64^ When using plasma p-tau217 as a screening tool to guide tau-PET scan referral, a significantly higher tau-PET positivity rate was observed across both the quantitative and visual read method.^14^ Given the similar results between the two methods and the ability of the visual read approach to detect tau deposition beyond the temporal lobe, including atypical patterns of tau accumulation, the authors highlight the clinical potential of this visual read method within their proposed workflow.^14^ Notably, this method rates isolated medial temporal lobe (MTL) uptake as positive, in contrast to the FDA-approved [^18^F]-flortaucipir visual read method, which classifies isolated MTL uptake as negative.

Future research including clinical data from diverse populations, large validation sets, and multi-reader assessments is needed to further validate visual read methods for tau-PET tracers such as [^18^F]RO948 and [^18^F]-MK6240, as well as to refine the approved visual read methods for [^18^F]-flortaucipir.^60,62,64^

### Differences in tau-PET tracers

The importance of structured visual read methods is underscored by off-target binding observed across tau-PET tracers. Different tau-PET tracers show distinct patterns of off-target binding. For instance, [^18^F]-flortaucipir exhibits off-target binding in regions such as the choroid plexus and basal ganglia, likely due to interactions with monoamine oxidase, melanin, and neuromelanin.^65–67^ Because this off-target binding interferes with the tau-PET signal in the hippocampus, this region is often excluded from analyses, despite its importance for early Braak staging.^1^ In contrast, second-generation tau-PET tracers, including [^18^F]-MK6240 and [^18^F]RO948, exhibit meningeal off-target binding.^68,69^ This meningeal signal can complicate quantification of tau-PET uptake in the MTL due to spill-in effects.^68^ Therefore, tracer-specific off-target binding should be considered and, where possible, corrected for in tau-PET quantification. Moreover, this highlights the value of visual read methods as a complementary approach to quantitative tau-PET assessment, particularly when off-target binding may complicate quantitative interpretation in key medial temporal regions.

The large variety of tau-PET tracers used in clinical research has prompted efforts to develop harmonization methods.^70–72^ One such effort is the CenTauR scale, which adopts a similar approach to the established Centiloid scale for Aβ-PET.^70,72^ However, this method requires a reference tracer, a process currently limited by the absence of a universally accepted gold-standard tau-PET tracer.^70^ Consequently, alternative approaches that do not rely on a reference tracer have been proposed, such as the use of a joint propagation model or the anchoring of SUVR values within and across tracers, termed the Uniτ scale, as implemented in the HEAD study.^70,71^ Head-to-head comparisons of different tracers across extensive multisite cohorts will be essential to further validate and expand these efforts.^70,71^

### Differences in plasma p-tau assays

Various plasma p-tau assays have been developed, differing primarily in the specific antibodies used and the analytical methods employed.^27^ Notably, these assays show varying degrees of association with tau-PET outcomes.^27^

In a head-to-head comparison, Warmenhoven et al. (2025) evaluated several plasma p-tau217 immunoassays, p-tau217_Lilly_, p-tau217_Janssen_, and p-tau217_ALZpath_, as well as a mass spectrometry assay (%p-tau217_WashU_), reflecting the ratio of phosphorylated to non-phosphorylated p-tau217 (Supplemental Table 1). While all plasma p-tau217 tests demonstrated high performance in detecting tau-PET positivity, plasma %p-tau217_WashU_ outperformed the immunoassays.^27^ Among the immunoassays, p-tau217_Lilly_ showed superior performance compared to p-tau217_ALZpath_ in identifying tau-PET status.^27^ With respect to baseline and longitudinal tau-PET load, plasma %p-tau217_WashU_ again showed the strongest associations.^27^ Among the immunoassays, p-tau217_Lilly_ outperformed both p-tau217_Janssen_ and p-tau217_ALZpath_.^27^ Based on these findings, the authors suggest that mass spectrometry assays may offer greater precision than immunoassays in detecting tau-PET abnormalities.^27^ Moreover, they discuss potential explanations for the differential performance of the immunoassays. For example, p-tau217_Janssen_ has been shown to also target p-tau212, potentially contributing to its slightly lower performance.^27,73^ In contrast, p-tau217_Lilly_ was designed not to target big tau, an isoform primarily released from peripheral sources, making it likely more specific to brain-derived tau.^27^

Despite the superior performance of certain plasma p-tau assays, these differences are often modest. Researchers therefore caution against overinterpreting the relative superiority of individual assays, particularly in light of the rapid development of assay technologies and the limited evaluation of their performance outside research settings.^14,27^ For instance, while mass spectrometry assays have demonstrated higher accuracy than immunoassays,^27^ their high cost and labor-intensive protocols may limit feasibility for clinical implementation. Nonetheless, studies using multicohort data should consider assay comparability, for instance by consulting previous head-to-head comparisons.^42^

### Target population: the importance of context

Another important consideration when interpreting plasma p-tau and tau-PET concordance is the context of the target population. As described earlier, greater concordance between these biomarkers is typically observed in symptomatic individuals compared to those in preclinical stages.^37,47,53^ More specifically, the negative and positive predictive values of these biomarkers likely depend on the prevalence of underlying pathology, the distribution of individuals across disease stages, and the frequency of comorbidities within the population.^26,27,34^ Therefore, biomarker thresholds should be interpreted within the specific context, ideally accounting for these additional factors.^26^ The importance of considering comorbidities and other biological confounders in the interpretation of plasma p-tau levels will be addressed in the following section.

## Biological confounders and comorbidities in plasma p-tau interpretation

Several comorbidities and other biological confounders have been shown to influence plasma p-tau levels.^1,74–76^ This susceptibility to systemic and biological confounders represents a key limitation of plasma biomarkers relative to PET imaging, which more directly reflects cortical tau pathology.^37^ Accounting for these factors is therefore critical to ensure accurate interpretation of plasma p-tau levels across diverse clinical and research settings.^76^ Insufficient consideration of comorbidities and other risk factors across study populations may lead to premature conclusions, for example, attributing variation in biomarker performance to specific plasma p-tau epitopes or assays, when such differences may, in part, reflect variation in the prevalence of biological confounders.^1^

### Demographics and risk factors in plasma p-tau

In a population-based study of individuals aged 30 to 98 years, Mielke et al. (2022) demonstrated that plasma p-tau217 and p-tau181 levels increased with age beginning between 65 and 70 years. This increase was particularly pronounced in Aβ-PET positive individuals.^76^ Specifically, the age-related rise in plasma p-tau217 was 12 times greater, and in plasma p-tau181 was 7.2 times greater, in Aβ-PET positive individuals compared to Aβ-PET negative individuals.^76^

With respect to sex, no significant differences were observed in plasma p-tau181 levels.^76^ However, plasma p-tau217 levels were higher in males than females, both in the overall cohort and among CU individuals.^76^ These sex differences were not observed among individuals with MCI or dementia.^76^ Notably, when individuals with chronic kidney disease (CKD), a history of stroke or myocardial infarction were excluded, the sex differences in plasma p-tau217 levels were attenuated, however, levels remained higher in males than in females.^76^

*APOE* ε4 status also demonstrated a significant association with plasma p-tau levels.^76^ Individuals carrying the *APOE* ε4 allele had significantly higher levels of plasma p-tau217 and p-tau181 compared to non-carriers, both in the overall cohort and among CU and MCI subgroups.^76^ This association is thought to reflect the increased risk of Aβ pathology among *APOE* ε4 carriers.^76^

### Kidney and liver function

Kidney and liver function are essential for proper metabolism and protein clearance, and both have been shown to influence plasma p-tau levels.^74,76^ For example, individuals with CKD exhibited significantly higher plasma p-tau217 and p-tau181 levels compared to those without CKD.^76^ Notably, the increase in plasma p-tau181 associated with CKD exceeded the difference observed between Aβ-PET positive and negative individuals.^76^ Berry et al. (2022) investigated a broader spectrum of hepatorenal dysfunction by examining associations between plasma p-tau181, creatinine, and albumin levels in individuals with cirrhosis. Plasma p-tau181 levels were two to four times higher in individuals with cirrhosis compared to controls.^74^ Among individuals with cirrhosis, creatinine, a marker of kidney function, where elevated levels indicate impairment, was moderately associated with higher plasma p-tau181 levels.^74^ In contrast, albumin, a marker of liver function, where lower levels indicate impairment, showed a weak inverse association with plasma p-tau181 in the same patients.^74^

Taken together, these findings suggest that conditions such as CKD and cirrhosis can lead to elevated levels of plasma p-tau217 and p-tau181 and should be considered potential confounders in the interpretation of plasma p-tau biomarkers in the context of AD.^74,76^

### Vascular comorbidities

In addition to the systemic conditions described, vascular comorbidities have also been associated with elevated levels of plasma p-tau217 and p-tau181.^76^ Individuals with a history of clinical stroke or myocardial infarction exhibited significantly higher plasma p-tau217 and p-tau181 levels compared to those without such histories.^76^ These associations remained significant after adjusting for Aβ-PET burden, suggesting they are not solely driven by Aβ pathology.^76^ After excluding individuals with CKD, a history of myocardial infarction remained significantly associated with higher plasma p-tau217 and p-tau181 levels, whereas the association with stroke was no longer significant.^76^ In addition, both higher white matter hyperintensity volumes and more silent brain infarcts have been associated with higher levels of plasma p-tau217 and p-tau181,^77,78^ underscoring the value of incorporating structural imaging markers when interpreting plasma p-tau results.

### Impact of comorbidities on plasma p-tau reference ranges and dementia prediction

Given the association between comorbidities and elevated plasma p-tau217 and p-tau181 levels, several studies have examined their impact on key outcomes such as reference ranges and dementia prediction.^75,76^ For instance, Mielke et al. (2022) compared plasma p-tau cut-offs derived from samples that either included or excluded individuals with CKD, a history of stroke, or myocardial infarction. When these individuals were excluded, the positivity cut-off for plasma p-tau181 decreased by 10%, and for plasma p-tau217 by 4%.^76^ Moreover, excluding individuals with these comorbidities enhanced the diagnostic performance of plasma p-tau, as reflected by higher Youden indices and areas under the ROC curve (AUROC) for tau-PET measures.^76^

In contrast, although creatinine and BMI were associated with plasma p-tau217 and p-tau181 levels, adjusting for these variables did not improve the predictive accuracy of plasma p-tau for conversion to AD dementia.^75^ However, studies specifically investigating the extent to which comorbidities and other biological confounders affect the clinical interpretation of plasma p-tau biomarkers remain limited.^75^ Therefore, further research is needed to determine which factors influence the establishment of reference ranges or prediction of disease progression beyond their association with elevated plasma p-tau levels.

### Future directions for plasma p-tau in the context of comorbidities

Another important direction for future research is to investigate the pathophysiological pathways through which comorbidities influence plasma p-tau levels.^76^ While factors such as *APOE* ε4 carriership likely affect plasma p-tau through increased risk of AD pathology, conditions such as CKD or cirrhosis may influence plasma p-tau levels through non-AD-related pathophysiological processes, including impaired protein clearance.^74,76^ The mechanisms underlying elevated plasma p-tau levels in individuals with a history of clinical stroke, silent brain infarcts, or myocardial infarction remain unclear.^76,78^ Preclinical experimental studies suggest that cerebral ischemia may induce tau hyperphosphorylation, while also disrupting blood-brain-barrier integrity and activating neuroinflammatory and oxidative stress pathways.^79,80^ These downstream inflammatory and oxidative processes may further amplify tau hyperphosphorylation.^79,80^ Collectively, these mechanisms may facilitate the release of phosphorylated tau into the circulation, thereby contributing to elevated plasma p-tau levels in individuals with vascular comorbidities. Understanding these pathways is essential for improving the interpretation of plasma p-tau biomarkers and minimizing false-positive results in individuals with comorbidities.^76^

Moreover, there is a need for analytical approaches that account for comorbidities and other risk factors known to influence plasma p-tau levels. Potential strategies include adjusting plasma p-tau levels for the presence of specific comorbidities or developing context-specific cut-offs.^76^ Additionally, recent findings suggest that ratios of phosphorylated tau to total tau, such as plasma p-tau217 to total tau217, may be less affected by certain comorbidities than plasma p-tau217 alone.^81^ Hence, implementing such ratio-based approaches, as also applied in the FDA-approved Lumipulse test (p-tau217/Aβ-42 plasma ratio),^45^ could enhance the specificity of plasma p-tau measurements for AD pathology in individuals with varying comorbidities and risk factors.^81^

Finally, it will be critical to include diverse populations in the development of plasma p-tau reference ranges.^82^ Given that the prevalence of comorbidities varies by ethnicity and socioeconomic status, examining plasma p-tau levels across diverse demographic groups will support the establishment of more accurate and generalizable reference standards.^76^

## Conclusion

This narrative review aimed to assess the concordance between plasma p-tau (p-tau217/p-tau181/p-tau231) positivity and tau-PET positivity. Three key conclusions emerge from the current literature.

First, the degree of concordance between plasma p-tau and tau-PET varies by disease stage, with lower concordance typically observed in preclinical compared to symptomatic stages.^37,47,53^ This early discordance, typically characterized by plasma p-tau positivity in the presence of tau-PET negativity, likely reflects differences in sensitivity to early versus advanced tau pathology.^37,42,53^ Specifically, plasma p-tau is thought to reflect a distinct pathophysiological stage of tau pathology, capturing soluble tau changes that precede aggregation into neurofibrillary tangles.^37^ While plasma p-tau217, p-tau181, and p-tau231 have all been associated with tau-PET SUVR values and tau-PET positivity,^1,17,37,47^ plasma p-tau217 has demonstrated superior performance in predicting tau-PET positivity.^26,31^

Second, accumulating evidence suggests a temporal sequence of plasma p-tau biomarker changes across the AD continuum.^2,33,37,53^ Specifically, plasma p-tau231 positivity appears to precede p-tau181 positivity, with increases in plasma p-tau231 levels observed even before Aβ-PET becomes positive.^33,37^ In contrast, while plasma p-tau181 is also thought to reflect early tau pathology, its levels have only been associated with tau-PET in Aβ-positive individuals.^2^ Additionally, plasma p-tau217 levels continue to rise with disease progression and have been linked to the spread of tau pathology beyond the medial temporal lobe.^29,53^ Since tau-PET positivity reflects more advanced stages of tau accumulation, these temporal dynamics may explain some of the variation in concordance across disease stages.^33,42,53^

To fully capture these dynamics, longitudinal studies are required for delineating the progression of plasma p-tau biomarkers from preclinical to symptomatic stages of AD.^33^ Such studies may also reveal more subtle plasma changes within specific disease stages.^33,83^ Ultimately, these efforts could lead to the development of a stage-specific trajectory framework, enabling quantification of individual plasma p-tau deviations. Given the influence of various comorbidities and other risk factors on plasma p-tau levels,^1,74–76^ these factors should be carefully considered when establishing such a normative framework. Furthermore, to ensure broad applicability, plasma p-tau reference standards should incorporate diverse demographic populations.^76,82^

Third, these findings have important implications for the clinical implementation of plasma p-tau and tau-PET. Improved quantification of individual plasma p-tau deviations is likely to enhance the efficacy of plasma p-tau biomarkers as screening tools. Previous studies have demonstrated the value of using plasma p-tau217 for tau-PET scan referrals or sample selection in clinical trials.^14,42^ Notably, plasma p-tau217 guided tau-PET scans offer significant prognostic value, and the sequential use of plasma p-tau217 followed by tau-PET can substantially reduce both costs and required sample size.^14,42^ These advantages may be even more pronounced with precise quantification of individual plasma p-tau deviations, enabling better identification of patients who would benefit most from tau-PET assessment of regional tau burden.^13,14^ Future research should aim to optimize the integration of plasma p-tau biomarkers and tau-PET across different clinical stages of AD.

Regarding this latter point, plasma p-tau biomarkers are generally expected to provide prognostic information primarily during the preclinical stages of AD, whereas tau-PET is likely more effective for monitoring clinical progression during symptomatic stages.^2,17,42^ Nonetheless, plasma p-tau217 and tau-PET have also been shown to provide complementary information on cognitive decline and conversion to MCI during the preclinical stage.^42^ Furthermore, elevated levels of plasma p-tau217 and p-tau181 have been associated with progression to MCI and AD.^34,35^ Thus, the predictive utility of plasma p-tau biomarkers and tau-PET for cognitive decline and disease progression likely varies depending on sample characteristics and clinical outcomes assessed, and warrants further investigation.

Recently, a novel plasma tau species, eMTBR-tau243, has been developed that specifically reflects insoluble tau aggregates characteristic of AD pathology and has shown strong associations with both tau-PET signal and cognitive performance.^23^ Preliminary findings suggest that plasma eMTBR-tau243 correlates more strongly with tau-PET than current p-tau biomarkers, offering potential as a biomarker for staging and diagnosis of AD.^23^ Notably, compared to plasma %p-tau217 and %p-tau181, eMTBR-tau243 demonstrates stronger associations with tau-PET signal in intermediate and late Braak regions, while showing comparable performance to %p-tau217 in early Braak regions.^23^ This suggests that eMTBR-tau243 may capture more advanced tau pathology that is less well reflected by existing plasma p-tau markers.^23^ Although further validation is required, eMTBR-tau243 holds promise as a next-generation plasma biomarker for tracking AD-related tau pathology.^23^

Another important consideration is the method used for tau-PET staging. While severity-based staging approaches and continuous tau-PET SUVR values are well-suited for research applications,^1,41^ visual read methods are more appropriate for clinical implementation.^60^ Currently, only [^18^F]-flortaucipir has received clinical approval, along with a corresponding visual read protocol.^43,44^ This visual read method emphasizes specificity, with a positive read corresponding to Braak stages V–VI pathology. Visual read methods for second-generation tau-PET tracers also show promise but still require further validation in large clinical and postmortem datasets.^14,60,62,64^ Furthermore, the development of tau-PET harmonization strategies, such as the CenTauR scale, joint propagation models, and Uniτ scale, will support better comparability across studies.^70–72^

Overall, the concordance between plasma p-tau positivity and tau-PET positivity varies according to the clinical stage of disease, the definition of tau-PET positivity, the plasma p-tau biomarker assessed, and the prevalence of comorbidities in the target population.^26,27,34,37,47,53,76^ Careful consideration of these factors will be essential for the optimal application of these biomarkers across diverse contexts, including AD diagnosis, clinical trial recruitment, treatment monitoring, and personalized patient care.^16,26,27,37^

## Supplemental Material

sj-docx-1-alz-10.1177_13872877261468708 - Supplemental material for Concordance between plasma biomarkers of phosphorylated tau and tau-PET: A narrative review Supplemental material, sj-docx-1-alz-10.1177_13872877261468708 for Concordance between plasma biomarkers of phosphorylated tau and tau-PET: A narrative review by Anna S. Van Houwelingen, Meike W. Vernooij, Marie R. Vermeiren, Rik Ossenkoppele, Elsmarieke Van De Giessen, Harro Seelaar and Julia Neitzel in Journal of Alzheimer's Disease

## References

[1] Jack CR Wiste HJ Algeciras-Schimnich A , et al. Predicting amyloid PET and tau PET stages with plasma biomarkers. Brain 2023; 146: 2029–2044. 36789483 10.1093/brain/awad042 PMC10151195

[2] Coomans EM Verberk IMW Ossenkoppele R , et al. A head-to-head comparison between plasma pTau181 and tau PET along the Alzheimer’s disease continuum. J Nucl Med 2023; 64: 437–443. 36229187 10.2967/jnumed.122.264279 PMC10071811

[3] Nelson PT Alafuzoff I Bigio EH , et al. Correlation of Alzheimer disease neuropathologic changes with cognitive status: a review of the literature. J Neuropathol Exp Neurol 2012; 71: 362–381. 22487856 10.1097/NEN.0b013e31825018f7 PMC3560290

[4] Wang Y Mandelkow E . Tau in physiology and pathology. Nat Rev Neurosci 2016; 17: 22–35. 26631930 10.1038/nrn.2015.1

[5] Teunissen CE Kolster R Triana-Baltzer G , et al. Plasma p-tau immunoassays in clinical research for Alzheimer’s disease. Alzheimers Dement 2025; 21: e14397. 10.1002/alz.14397 PMC11977406 39625101

[6] Groot C Smith R Collij LE , et al. Tau positron emission tomography for predicting dementia in individuals with mild cognitive impairment. JAMA Neurol 2024; 81: 845–856. 38857029 10.1001/jamaneurol.2024.1612 PMC11165418

[7] Ossenkoppele R Rabinovici GD Smith R , et al. Discriminative accuracy of [18F]flortaucipir positron emission tomography for Alzheimer disease vs other neurodegenerative disorders. JAMA 2018; 320: 1151–1162. 30326496 10.1001/jama.2018.12917 PMC6233630

[8] Ossenkoppele R Pichet Binette A Groot C , et al. Amyloid and tau PET-positive cognitively unimpaired individuals are at high risk for future cognitive decline. Nat Med 2022; 28: 2381–2387. 36357681 10.1038/s41591-022-02049-x PMC9671808

[9] Mormino EC Toueg TN Azevedo C , et al. Tau PET imaging with 18F-PI-2620 in aging and neurodegenerative diseases. Eur J Nucl Med Mol Imaging 2021; 48: 2233–2244. 32572562 10.1007/s00259-020-04923-7 PMC7755737

[10] Pascoal TA Therriault J Benedet AL , et al. 18F-MK-6240 PET for early and late detection of neurofibrillary tangles. Brain 2020; 143: 2818–2830. 32671408 10.1093/brain/awaa180

[11] La Joie R Visani AV Baker SL , et al. Prospective longitudinal atrophy in Alzheimer’s disease correlates with the intensity and topography of baseline tau-PET. Sci Transl Med 2020; 12: eaau5732. 10.1126/scitranslmed.aau5732 PMC7035952 31894103

[12] Ossenkoppele R Schonhaut DR Baker SL , et al. Tau, amyloid, and hypometabolism in a patient with posterior cortical atrophy. Ann Neurol 2015; 77: 338–342. 25448043 10.1002/ana.24321 PMC4382124

[13] Vogel JW Young AL Oxtoby NP , et al. Four distinct trajectories of tau deposition identified in Alzheimer’s disease. Nat Med 2021; 27: 871–881. 33927414 10.1038/s41591-021-01309-6 PMC8686688

[14] Brum WS Cullen NC Therriault J , et al. A blood-based biomarker workflow for optimal tau-PET referral in memory clinic settings. Nat Commun 2024; 15: 2311. 38486040 10.1038/s41467-024-46603-2 PMC10940585

[15] Ossenkoppele R Hansson O . Towards clinical application of tau PET tracers for diagnosing dementia due to Alzheimer’s disease. Alzheimers Dement 2021; 17: 1998–2008. 33984177 10.1002/alz.12356

[16] Hansson O Blennow K Zetterberg H , et al. Blood biomarkers for Alzheimer’s disease in clinical practice and trials. Nat Aging 2023; 3: 506–519. 37202517 10.1038/s43587-023-00403-3 PMC10979350

[17] Mundada NS Rojas JC Vandevrede L , et al. Head-to-head comparison between plasma p-tau217 and flortaucipir-PET in amyloid-positive patients with cognitive impairment. Alzheimers Res Ther 2023; 15: 157. 37740209 10.1186/s13195-023-01302-w PMC10517500

[18] Ashton NJ Puig-Pijoan A Milà-Alomà M , et al. Plasma and CSF biomarkers in a memory clinic: head-to-head comparison of phosphorylated tau immunoassays. Alzheimers Dement 2023; 19: 1913–1924. 36370462 10.1002/alz.12841 PMC10762642

[19] Blennow K Hampel H Weiner M , et al. Cerebrospinal fluid and plasma biomarkers in Alzheimer disease. Nat Rev Neurol 2010; 6: 131–144. 20157306 10.1038/nrneurol.2010.4

[20] Janelidze S Mattsson N Palmqvist S , et al. Plasma P-tau181 in Alzheimer’s disease: relationship to other biomarkers, differential diagnosis, neuropathology and longitudinal progression to Alzheimer’s dementia. Nat Med 2020; 26: 379–386. 32123385 10.1038/s41591-020-0755-1

[21] Palmqvist S Janelidze S Quiroz YT , et al. Discriminative accuracy of plasma phospho-tau217 for Alzheimer disease vs other neurodegenerative disorders. JAMA 2020; 324: 772–781. 32722745 10.1001/jama.2020.12134 PMC7388060

[22] Gonzalez-Ortiz F Turton M Kac PR , et al. Brain-derived tau: a novel blood-based biomarker for Alzheimer’s disease-type neurodegeneration. Brain 2022; 146: 1152–1165. 10.1093/brain/awac407 PMC9976981 36572122

[23] Horie K Salvadó G Koppisetti RK , et al. Plasma MTBR-tau243 biomarker identifies tau tangle pathology in Alzheimer’s disease. Nat Med 2025; 31: 2044–2053. 40164726 10.1038/s41591-025-03617-7 PMC12176612

[24] Jack CR Jr Andrews JS Beach TG , et al. Revised criteria for diagnosis and staging of Alzheimer’s disease: Alzheimer’s association workgroup. Alzheimers Dement 2024; 20: 5143–5169. 38934362 10.1002/alz.13859 PMC11350039

[25] Montoliu-Gaya L Benedet AL Tissot C , et al. Mass spectrometric simultaneous quantification of tau species in plasma shows differential associations with amyloid and tau pathologies. Nat Aging 2023; 3: 661–669. 37198279 10.1038/s43587-023-00405-1 PMC10275761

[26] Ashton NJ Brum WS Di Molfetta G , et al. Diagnostic accuracy of a plasma phosphorylated tau 217 immunoassay for Alzheimer disease pathology. JAMA Neurol 2024; 81: 255–263. 38252443 10.1001/jamaneurol.2023.5319 PMC10804282

[27] Warmenhoven N Salvadó G Janelidze S , et al. A comprehensive head-to-head comparison of key plasma phosphorylated tau 217 biomarker tests. Brain 2025; 148: 416–431. 39468767 10.1093/brain/awae346 PMC11788211

[28] Janelidze S Bali D Ashton NJ , et al. Head-to-head comparison of 10 plasma phospho-tau assays in prodromal Alzheimer’s disease. Brain 2023; 146: 1592–1601. 36087307 10.1093/brain/awac333 PMC10115176

[29] Ashton NJ Janelidze S Mattsson-Carlgren N , et al. Differential roles of Aβ42/40, p-tau231 and p-tau217 for Alzheimer’s trial selection and disease monitoring. Nat Med 2022; 28: 2555–2562. 36456833 10.1038/s41591-022-02074-w PMC9800279

[30] Leuzy A Janelidze S Mattsson-Carlgren N , et al. Comparing the clinical utility and diagnostic performance of CSF P-Tau181, P-Tau217, and P-Tau231 assays. Neurology 2021; 97: e1681–e1694. 10.1212/WNL.0000000000012727 PMC8605616 34493616

[31] Thijssen EH La Joie R Strom A , et al. Plasma phosphorylated tau 217 and phosphorylated tau 181 as biomarkers in Alzheimer’s disease and frontotemporal lobar degeneration: a retrospective diagnostic performance study. Lancet Neurol 2021; 20: 739–752. 34418401 10.1016/S1474-4422(21)00214-3 PMC8711249

[32] Jonaitis EM Janelidze S Cody KA , et al. Plasma phosphorylated tau 217 in preclinical Alzheimer’s disease. Brain Commun 2023; 5: fcad057. 10.1093/braincomms/fcad057 PMC10066514 37013174

[33] Ashton NJ Pascoal TA Karikari TK , et al. Plasma p-tau231: a new biomarker for incipient Alzheimer’s disease pathology. Acta Neuropathol 2021; 141: 709–724. 33585983 10.1007/s00401-021-02275-6 PMC8043944

[34] Palmqvist S Tideman P Cullen N , et al. Prediction of future Alzheimer’s disease dementia using plasma phospho-tau combined with other accessible measures. Nat Med 2021; 27: 1034–1042. 34031605 10.1038/s41591-021-01348-z

[35] Simrén J Leuzy A Karikari TK , et al. The diagnostic and prognostic capabilities of plasma biomarkers in Alzheimer’s disease. Alzheimers Dement 2021; 17: 1145–1156. 33491853 10.1002/alz.12283 PMC8359457

[36] Ossenkoppele R van der Kant R Hansson O . Tau biomarkers in Alzheimer’s disease: towards implementation in clinical practice and trials. Lancet Neurol 2022; 21: 726–734. 35643092 10.1016/S1474-4422(22)00168-5

[37] Tissot C Therriault J Kunach P , et al. Comparing tau status determined via plasma pTau181, pTau231 and [(18)F]MK6240 tau-PET. EBioMedicine 2022; 76: 103837. 35134647 10.1016/j.ebiom.2022.103837 PMC8844756

[38] Sintini I Graff-Radford J Senjem ML , et al. Longitudinal neuroimaging biomarkers differ across Alzheimer’s disease phenotypes. Brain 2020; 143: 2281–2294. 32572464 10.1093/brain/awaa155 PMC7363492

[39] Therriault J Vermeiren M Servaes S , et al. Association of phosphorylated tau biomarkers with amyloid positron emission tomography vs tau positron emission tomography. JAMA Neurol 2023; 80: 188–199. 36508198 10.1001/jamaneurol.2022.4485 PMC9856704

[40] Barthélemy NR Li Y Joseph-Mathurin N , et al. A soluble phosphorylated tau signature links tau, amyloid and the evolution of stages of dominantly inherited Alzheimer’s disease. Nat Med 2020; 26: 398–407. 32161412 10.1038/s41591-020-0781-z PMC7309367

[41] Mattsson-Carlgren N Leuzy A Janelidze S , et al. The implications of different approaches to define AT(N) in Alzheimer disease. Neurology 2020; 94: e2233–e2244. 10.1212/WNL.0000000000009485 PMC7357296 32398359

[42] Ossenkoppele R Salvadó G Janelidze S , et al. Plasma p-tau217 and tau-PET predict future cognitive decline among cognitively unimpaired individuals: implications for clinical trials. Nat Aging 2025; 5: 883–896. 40155777 10.1038/s43587-025-00835-z PMC12092243

[43] Eli Lilly and Company. Highlights of prescribing information. Eli Lilly and Company, https://www.accessdata.fda.gov/drugsatfda_docs/label/2020/212123s000lbl.pdf#:∼:text=stable%20oxygen%2018%20with%20a,Table%203 (2020).

[44] European Medicines Agency (EMA). Tauvid | European Medicines Agency (EMA). https://www.ema.europa.eu/en/medicines/human/EPAR/tauvid (2024).

[45] U.S. Food and Drug Administration. FDA clears first blood test used in diagnosing Alzheimer’s disease, https://www.fda.gov/news-events/press-announcements/fda-clears-first-blood-test-used-diagnosing-alzheimers-disease (2025).

[46] Therriault J Pascoal TA Benedet AL , et al. Frequency of biologically defined Alzheimer disease in relation to age, sex, APOE ε4, and cognitive impairment. Neurology 2021; 96: e975–e985. 10.1212/WNL.0000000000011416 PMC8055338 33443136

[47] Ferreira PCL Therriault J Tissot C , et al. Plasma p-tau231 and p-tau217 inform on tau tangles aggregation in cognitively impaired individuals. Alzheimers Dement 2023; 19: 4463–4474. 37534889 10.1002/alz.13393 PMC10592380

[48] Lowe VJ Lundt ES Albertson SM , et al. Tau-positron emission tomography correlates with neuropathology findings. Alzheimers Dement 2020; 16: 561–571. 31784374 10.1016/j.jalz.2019.09.079 PMC7067654

[49] Leuzy A Smith R Ossenkoppele R , et al. Diagnostic performance of RO948 F 18 tau positron emission tomography in the differentiation of Alzheimer disease from other neurodegenerative disorders. JAMA Neurol 2020; 77: 955–965. 32391858 10.1001/jamaneurol.2020.0989 PMC7215644

[50] Vogel JW Iturria-Medina Y Strandberg OT , et al. Spread of pathological tau proteins through communicating neurons in human Alzheimer’s disease. Nat Commun 2020; 11: 2612. 32457389 10.1038/s41467-020-15701-2 PMC7251068

[51] Ercan-Herbst E Ehrig J Schöndorf DC , et al. A post-translational modification signature defines changes in soluble tau correlating with oligomerization in early stage Alzheimer’s disease brain. Acta Neuropathol Commun 2019; 7: 192. 31796124 10.1186/s40478-019-0823-2 PMC6892178

[52] Neddens J Temmel M Flunkert S , et al. Phosphorylation of different tau sites during progression of Alzheimer’s disease. Acta Neuropathol Commun 2018; 6: 52. 29958544 10.1186/s40478-018-0557-6 PMC6027763

[53] Mattsson-Carlgren N Janelidze S Bateman RJ , et al. Soluble P-tau217 reflects amyloid and tau pathology and mediates the association of amyloid with tau. EMBO Mol Med 2021; 13: e14022. 10.15252/emmm.202114022 PMC8185545 33949133

[54] Medina M Avila J . Further understanding of tau phosphorylation: implications for therapy. Expert Rev Neurother 2015; 15: 115–122. 25555397 10.1586/14737175.2015.1000864

[55] Basheer N Smolek T Hassan I , et al. Does modulation of tau hyperphosphorylation represent a reasonable therapeutic strategy for Alzheimer’s disease? From preclinical studies to the clinical trials. Mol Psychiatry 2023; 28: 2197–2214. 37264120 10.1038/s41380-023-02113-z PMC10611587

[56] Fleisher AS Pontecorvo MJ Devous MD Sr , et al. Positron emission tomography imaging with [18F]flortaucipir and postmortem assessment of Alzheimer disease neuropathologic changes. JAMA Neurol 2020; 77: 829–839. 32338734 10.1001/jamaneurol.2020.0528 PMC7186920

[57] Therriault J Janelidze S Benedet AL , et al. Diagnosis of Alzheimer’s disease using plasma biomarkers adjusted to clinical probability. Nature Aging 2024; 4: 1529–1537. 39533113 10.1038/s43587-024-00731-y PMC11564087

[58] Schindler SE Galasko D Pereira AC , et al. Acceptable performance of blood biomarker tests of amyloid pathology - recommendations from the global CEO initiative on Alzheimer’s disease. Nat Rev Neurol 2024; 20: 426–439. 38866966 10.1038/s41582-024-00977-5

[59] Palmqvist S Tideman P Mattsson-Carlgren N , et al. Blood biomarkers to detect Alzheimer disease in primary care and secondary care. JAMA 2024; 332: 1245–1257. 39068545 10.1001/jama.2024.13855 PMC11284636

[60] Tunali I Wang J Arora AK , et al. Development and validation of a (18)F-flortaucipir PET visual stratification method. J Nucl Med 2025; 66: 612–619. 40081955 10.2967/jnumed.124.268700 PMC11960607

[61] Leuzy A Bollack A Pellegrino D , et al. Considerations in the clinical use of amyloid PET and CSF biomarkers for Alzheimer’s disease. Alzheimers Dement 2025; 21: e14528. 10.1002/alz.14528 PMC11881640 40042435

[62] Shuping JL Matthews DC Adamczuk K , et al. Development, initial validation, and application of a visual read method for [(18)F]MK-6240 tau PET. Alzheimers Dement (N Y) 2023; 9: e12372. 10.1002/trc2.12372 PMC9983143 36873926

[63] Coomans EM de Koning LA Rikken RM , et al. Performance of a [(18)F]flortaucipir PET visual read method across the Alzheimer disease continuum and in dementia with Lewy bodies. Neurology 2023; 101: e1850–e1862. 10.1212/WNL.0000000000207794 PMC10663007 37748892

[64] Smith R Hägerström D Pawlik D , et al. Clinical utility of tau positron emission tomography in the diagnostic workup of patients with cognitive symptoms. JAMA Neurol 2023; 80: 749–756. 37213093 10.1001/jamaneurol.2023.1323 PMC10203972

[65] Marquié M Normandin MD Vanderburg CR , et al. Validating novel tau positron emission tomography tracer [F-18]-AV-1451 (T807) on postmortem brain tissue. Ann Neurol 2015; 78: 787–800. 26344059 10.1002/ana.24517 PMC4900162

[66] Hostetler ED Walji AM Zeng Z , et al. Preclinical characterization of 18F-MK-6240, a promising PET tracer for in vivo quantification of human neurofibrillary tangles. J Nucl Med 2016; 57: 1599–1606. 27230925 10.2967/jnumed.115.171678

[67] Lowe VJ Curran G Fang P , et al. An autoradiographic evaluation of AV-1451 tau PET in dementia. Acta Neuropathol Commun 2016; 4: 58. 27296779 10.1186/s40478-016-0315-6 PMC4906968

[68] McVea A DiFilippo A McLachlan MJ , et al. Evaluating the effect of extra-cerebral off-target binding in [F-18]MK6240 PET scans in early-stage Alzheimer’s disease. Imaging Neurosci (Camb) 2024; 2: imag-2–00135. 10.1162/imag_a_00135 PMC12247575 40800261

[69] Smith R Strandberg O Leuzy A , et al. Sex differences in off-target binding using tau positron emission tomography. Neuroimage Clin 2021; 31: 102708. 34091353 10.1016/j.nicl.2021.102708 PMC8182304

[70] Leuzy A Raket LL Villemagne VL , et al. Harmonizing tau positron emission tomography in Alzheimer’s disease: the CenTauR scale and the joint propagation model. Alzheimers Dement 2024; 20: 5833–5848. 39041435 10.1002/alz.13908 PMC11497758

[71] Povala G Bauer-Negrini G Bellaver B , et al. Universal tau PET scale (Uniτ) – the HEAD study. Alzheimers Dement 2024; 20: e094383.

[72] Villemagne VL Leuzy A Bohorquez SS , et al. Centaur: toward a universal scale and masks for standardizing tau imaging studies. Alzheimers Dement (Amst) 2023; 15: e12454. 10.1002/dad2.12454 PMC10326476 37424964

[73] Triana-Baltzer G Moughadam S Slemmon R , et al. Development and validation of a high-sensitivity assay for measuring p217+tau in plasma. Alzheimers Dement (Amst) 2021; 13: e12204. 10.1002/dad2.12204 PMC8158165 34095436

[74] Berry K Asken BM Grab JD , et al. Hepatic and renal function impact concentrations of plasma biomarkers of neuropathology. Alzheimers Dement (Amst) 2022; 14: e12321. 10.1002/dad2.12321 PMC9274803 35845260

[75] Pichet Binette A Janelidze S Cullen N , et al. Confounding factors of Alzheimer’s disease plasma biomarkers and their impact on clinical performance. Alzheimers Dement 2023; 19: 1403–1414. 36152307 10.1002/alz.12787 PMC10499000

[76] Mielke MM Dage JL Frank RD , et al. Performance of plasma phosphorylated tau 181 and 217 in the community. Nat Med 2022; 28: 1398–1405. 35618838 10.1038/s41591-022-01822-2 PMC9329262

[77] Mielke MM Frank RD Dage JL , et al. Comparison of plasma phosphorylated tau species with amyloid and tau positron emission tomography, neurodegeneration, vascular pathology, and cognitive outcomes. JAMA Neurol 2021; 78: 1108–1117. 34309632 10.1001/jamaneurol.2021.2293 PMC8314178

[78] Gunasekaran TI Sanchez D Reyes-Dumeyer D , et al. Frequency of microvascular pathology and hippocampal atrophy on magnetic resonance imaging in a community study of Alzheimer’s disease with blood-based biomarkers. Ann Neurol 2025; 98: 1027–1043. 40735976 10.1002/ana.70006 PMC12577681

[79] Yang L-Y Hsu C-H Cheng Y-W , et al. Antroquinonol mitigates Tau hyperphosphorylation, neuronal damage and cognitive impairments in a chronic cerebral ischemia rat model. Biomed Pharmacother 2025; 189: 118271. 40544750 10.1016/j.biopha.2025.118271

[80] Pluta R Ułamek-Kozioł M Januszewski S , et al. Tau protein dysfunction after brain ischemia. J Alzheimers Dis 2018; 66: 429–437. 30282370 10.3233/JAD-180772 PMC6218135

[81] Janelidze S Barthélemy NR He Y , et al. Mitigating the associations of kidney dysfunction with blood biomarkers of Alzheimer disease by using phosphorylated tau to total tau ratios. JAMA Neurol 2023; 80: 516–522. 36987840 10.1001/jamaneurol.2023.0199 PMC10061310

[82] Manrai AK Patel CJ Ioannidis JPA . In the era of precision medicine and big data, who is normal? JAMA 2018; 319: 1981–1982. 29710130 10.1001/jama.2018.2009 PMC7572221

[83] Horie K Barthélemy NR Mallipeddi N , et al. Regional correlation of biochemical measures of amyloid and tau phosphorylation in the brain. Acta Neuropathol Commun 2020; 8: 149. 32854776 10.1186/s40478-020-01019-z PMC7450927

