# Supplementary material for: Concordance between plasma biomarkers of phosphorylated tau and tau-PET: A narrative review
Source: J Alzheimers Dis. 2026 Aug 8;113(2):561–77. doi: 10.1177/13872877261468708 (PMC13554363; doi:10.1177/13872877261468708)
Supplement: Supplementary file 1 [file sj-docx-1-alz-10.1177_13872877261468708.docx]

**Supplemental Material**

**Concordance between plasma biomarkers of phosphorylated tau and tau-PET: A narrative review**

**Supplementary Table 1.** Summary of plasma p-tau217 assays.

| Plasma p-tau217 assay | Platform | Distinguishing features |
| --- | --- | --- |
| Immunoassays |  |  |
| p-tau217_Lilly_ | Meso Scale Discovery | Designed not to target big tau^1^ |
| p-tau217_Janssen_ | Simoa | Also targets p-tau212^1, 2^ |
| p-tau217_ALZpath_ | Simoa | Uses a monoclonal capture antibody^1^ |
| Mass spectrometry assay | | |
| %p-tau217_WashU_ | NanoLC-MS/HRMS | Measures ratio of phosphorylated to non-phosphorylated tau^1^ |

Summary of the plasma p-tau217 assays investigated by Warmenhoven et al. (2025). The table includes the immunoassays and mass spectrometry assay, the platforms used, and key distinguishing features. Simoa: Single Molecule Array; NanoLC-MS/HRMS: Nano-Liquid Chromatography tandem High-Resolution Mass Spectrometry.

**References**

1.	Warmenhoven N, Salvadó G, Janelidze S, et al. A comprehensive head-to-head comparison of key plasma phosphorylated tau 217 biomarker tests. *Brain* 2025; 148: 416-431.

2.	Triana-Baltzer G, Moughadam S, Slemmon R, et al. Development and validation of a high-sensitivity assay for measuring p217+tau in plasma. *Alzheimers Dement (Amst)* 2021; 13: e12204.
